# Arsenic removal from aqueous solutions by diethylenetriamine-functionalized resin: isotherm, kinetics, selectivity and mechanism

**DOI:** 10.1098/rsos.181013

**Published:** 2018-09-12

**Authors:** Juncai Zhang, Youning Chen, Wei Zhao, Yuhong Li

**Affiliations:** College of Chemistry and Chemical Engineering, Xianyang Normal College, Xianyang 712000, People's Republic of China

**Keywords:** chloromethylated polystyrene bead, surface-initiated atom transfer radical polymerization, diethylenetriamine-functionalized resin, adsorption

## Abstract

The surface-initiated atom transfer radical polymerization method was used to graft glycidyl methacrylate (GMA) on chloromethylated cross-linked styrene-divinylbenzene resin, and then the novel diethylenetriamine-functionalized resin was prepared through the amination reaction between amino group of diethylenetriamine and epoxy group in GMA. The adsorption properties were evaluated with As(V). The adsorption of As(V) was mainly regarded as the electrostatic interaction between the adsorbent and the adsorbate by analysing the relationship between adsorption capacity with the solution pH, adsorption isotherm and adsorption kinetics. The maximum sorption capacity of As(V) was 5.25 mmol g^−1^. The adsorption isotherms of As(V) were best described by the Langmuir model, and its adsorption kinetics followed the pseudo-second-order kinetic equation. The adsorption of As(V) ions was hardly affected by common coexisting ions such as Na(I), K(I), Ca(II) and Mg(II), whereas it was slightly decreased when Fe(II) and Zn(II) coexisted in the solution, which illustrates the selective adsorption of As(V) from wastewater. Ten adsorption–desorption cycles demonstrated that the resin possessed high recycling efficiency and stability and was suitable for efficient removal of metal ions from aqueous solution.

## 1. Introduction

Heavy metal pollution is one of the most important environmental problems today. Chelating resin, a kind of functional polymer material, can form a coordination complex with metal ions, with the function of separation and enrichment for heavy metal ions, and is widely used in the field of heavy metal waste water treatment [1–6]. The density of functional groups on adsorbent surface is one of the factors affecting the sorption capacity. Some low-molecular-weight chemicals are often used to functionalize the material surface, but the adsorption capacity is not very satisfactory due to the limited adsorption sites on the material surface. This results in a low process economy for the removal of heavy metal ions. A number of studies have shown that polymers containing functional groups can enhance the adsorption capacity due to the polymers' highly complex-forming capabilities with metal ions. Thus, much attention has been given to grafting linear polymers onto resin particles [7–10]. As is well known, there are several methods for preparing grafting polymers, such as the chemisorption of a reactive polymer end group to the surface [11], grafting a polymer chain through a monomer covalently linked to the surface [12] and grafting a polymer chain from a surface modified with polymerization initiators [13]. Of these methods, maximum structural control can be achieved by the ‘grafting from’ route [14,15]. One of the popular living/controlled techniques, atom transfer radical polymerization (ATRP), can be used for the preparation of well-defined polymer brushes with dormant chain ends on various types of substrates [16,17]. In particular, ATRP has attracted considerable attention as a method for the preparation of functional materials because of its excellent properties: the precise synthesis of macromolecules with predetermined molecular weights, a designed molecular weight distribution, and tunable topology, composition and functionality. Our group prepared several chelating resins via ATRP, confirming that ATRP is one of the new methods for preparation of high capacity adsorption material [18–20].

Because the amino group has strong chelating properties for transition metal ions, adsorbents with amino groups have been widely used for removing heavy metal ions from solution [13,21–24]. In this paper, a novel diethylenetriamine-functionalized resin (DETA-PS resin) was prepared by surface-initiated ATRP (SI-ATRP) of glycidyl methacrylate (GMA) and the amination reaction between epoxy group and amino group of diethylenetriamine and epoxy group in GMA. The adsorption performance for As(V) was studied, which may provide a sort of efficient adsorbent for the treatment of heavy metal wastewater.

## 2. Experiment

### 2.1. Reagents and instrument

Chloromethylated polystyrene (Xi'an Lanxiao Science and Technology New Material Co., Ltd, with a chlorinate of 18% (mass fraction)); GMA (Aladdin, Shanghai, China); 2,2-dipyridyl (Sinopharm Chemical Reagent Co., Ltd, China); cuprous bromide (Tianjin no. 1 Chemical Reagent Factory, China); diethylenetriamine (Aladdin, Shanghai, China). All other chemicals were of analytical grade.

The concentrations of metal ions were measured by an atomic absorption spectrophotometry (AAS) instrument (novAA400, Analytic Jena AG, Germany).

### 2.2. Preparation of diethylenetriamine-functionalized resin

(1) GMA was grafted onto the surface of the initiator-functionalized resins via SI-ATRP. Briefly, 2,2′-bipyridyl (0.5 g, 0.72 mmol), copper(I) bromide (0.05 g, 0.30 mmol) and CMPS beads (5.0 g) were placed in a reaction vessel, and the mixture was de-oxygenated by repeated vacuuming. High-purity nitrogen was introduced into the tube after each evacuation stage. Then, GMA (10 ml) and 30 ml of tetrahydrofuran, de-oxygenated by three cycles of freezing–vacuum–thaw, were added into the tube using a syringe under a nitrogen atmosphere. The reaction was stirred at 40°C under a nitrogen atmosphere. The beads were filtered out from the reaction solution after the desired reaction time, and then immersed in 60 ml of a methanol/EDTANa_2_ (1 : 1, v/v) solution for 24 h to remove copper. Finally, the resulting GMA-modified PS (PGMA-g-PS) was washed by water and methanol and dried at 35°C under vacuum for the next reaction.
(2) 5 g of the modified PGMA-g-PS beads, 6.0 g of diethylenetriamine and 50 ml of tetrahydrofuran were added to a three-necked flask under stirring for 30 min. The pH of the mixture was adjusted to 12 and stirred at 50°C for 12 h. The resulting DETA-PS resins were filtered and washed thoroughly with ethanol and deionized water in sequence, and finally dried at 35°C in a vacuum oven.

### 2.3. Characterization of resin

X-ray photoelectron spectroscopy (XPS; PE, PHI-5400, USA) was used for the characterization of CMPS, PGMA-g-PS and DETA-PS resin.

### 2.4. Adsorption feature of resin

An estimated 0.100 g of DETA-PS resin was added into a series of 100 ml of 7 mmol l^−1^ As(V) solutions with different pH values, and the mixtures were shaken at 200 r.p.m. for 12 h at 25°C. The resins were removed by filtration, and the filtrates were collected to measure the final concentration of metal ion by AAS. The amount of metal ion adsorbed was calculated according to equation (2.1):

2.1 $$Q=\frac{(C_{}-C_{e})V}{W},$$

where *Q*_e_ is the equilibrium adsorption capacity (mmol g^−1^); *C*_0_ and *C*_e_ are the initial and equilibrium metal ion concentrations (mmol l^−1^), respectively; *V* is the solution volume (l); and *W* is the mass of the dried resins (g).

The adsorption capacities of DETA-PS resin at given temperatures were measured using the following procedure. As(V) stock solutions were adjusted to pH 4.0, and then diluted with NaAc–HAc solution (pH 4.0) to get a series of working solutions with different concentrations (0.5– 9.0 mmol l^−1^). 0.1 g of PVT-g-PS beads were added into 100.00 ml of operational solution, and then shaken on a shaker for 12 h. The beads were removed by filtration, and the filtrates were collected to measure the final ion concentration by AAS. Adsorption isotherms were obtained by plotting *Q*_e_ versus *C*_e_.

To a series of 0.1 g of diethylenetriamine chelating resin, 100.00 ml of 7.0 mmol l^−1^ As(V) working solutions were added in vessel, and then the solutions were shaken on a shaker at 25°C. Aliquots of 1.00 ml solution were taken at different time intervals, and the concentration variations of metal ion were analysed by AAS. The kinetic curve was obtained by a plot of *Q*_e_ versus adsorption time.

### 2.5. Adsorption selectivity

The adsorption selectivity of the resin was investigated under competitive conditions, and approximately 0.1 g of the resin was placed in contact with 100 ml of a binary mixture system (pH 4.0) in which the concentration of As(V) ion was 7.0 mmol l^−1^ and was half of the coexisting ions. The mixture was shaken for 12 h at 25°C. The selectivity factor was defined as the ratio of adsorption capacities of metal ions in the binary mixture.

### 2.6. Recycling experiments

The As(V)-loaded resins were exposed to 0.1 mol l^−1^ NaOH solution to be regenerated for 24 h and then were collected from the solutions by filtration at 35°C. The regenerated resin was re-used in the next cycle of adsorption experiments. The adsorption–desorption experiments were conducted at room temperature for 10 cycles.

## 3. Results and discussion

### 3.1. Characterization of resin

**Scheme 1.**
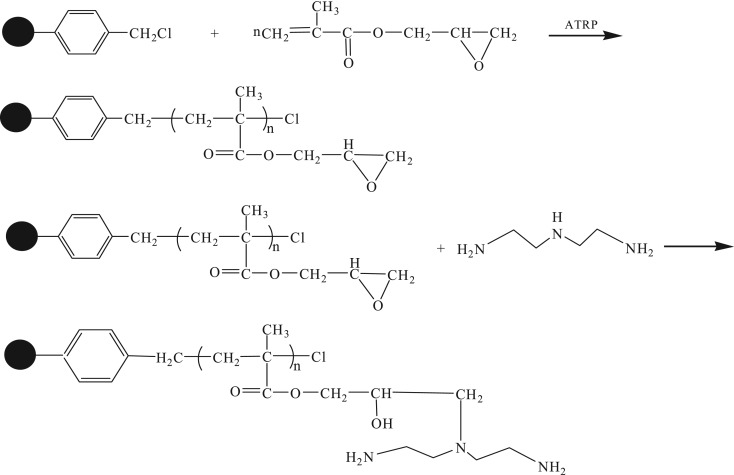
Synthetic route for the preparation of DETA-PS resin.

The two-step method was adopted for the synthesis of DETA-PS resin, as shown in scheme 1. In the first step, PGMA was grafted onto the surface of CMPS beads by SI-ATRP. In the second step, the amino groups were introduced into the polymer chain by amination reaction between amino group of diethylenetriamine and epoxy group in GMA.

**Figure 1.**
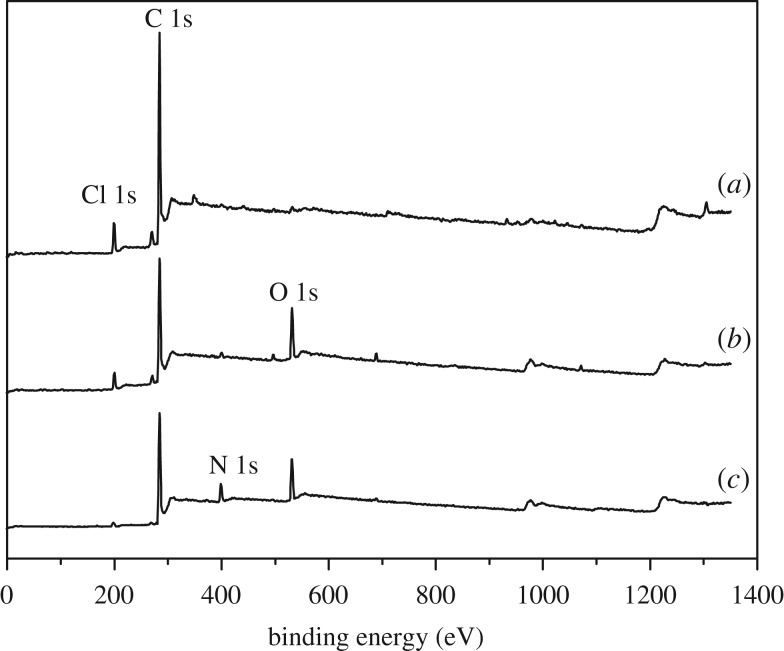
XPS wide scans of (*a*) CMPS, (*b*) PGMA-g-PS and (c) DETA-PS resin.

The chemical compositions of the original chloromethylated CMPS, PGMA-g-PS and DETA-PS resins were characterized by XPS. By analysing the wide-scan XPS spectra (figure 1), we could easily find the chemical change in the structure of the modified resins. The peak at 532.5 eV is due to O 1s (figure 1*b*), suggesting that the GMA is introduced into surface of the resin via SI-ATRP; after ring-opening reaction, the appearance of the peak of N 1s at 396.4 eV shows that the diethylenetriamine has been successfully introduced into the PGMA-g-PS resin (figure 1*c*). Overall, all the results indicated the GMA has been successfully grafted to the PS resin via SI-ATRP, and the amino group is introduced into the molecule chain.

**Figure 2.**
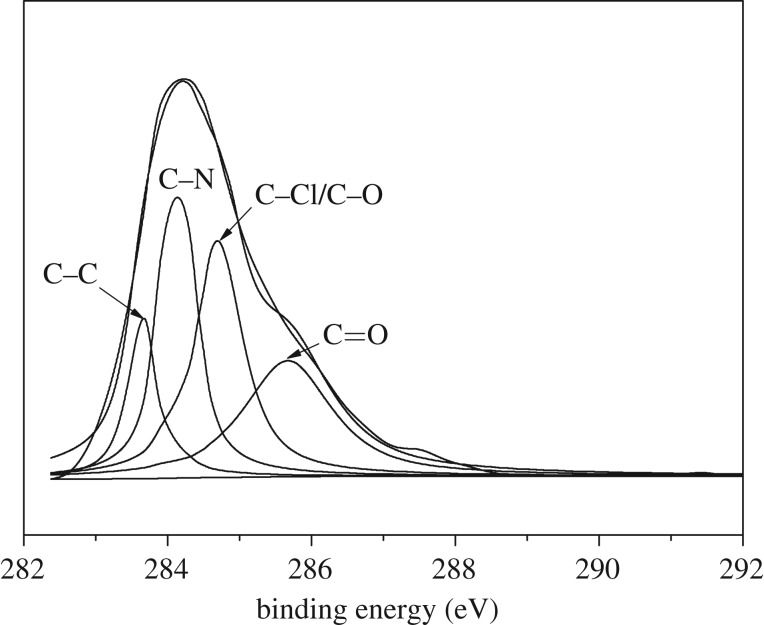
C 1s core-level spectrum of DETA-PS resin.

To obtain more detailed information about the change in the composition of the surface of DETA-PS resin, the peak components of the C 1s peak in the XPS spectra were further analysed in the core-level spectrum (figure 2). The C 1s core-level spectrum of the DETA-PS resin could be curve-fitted into four peak components. Peaks with binding energies at 283.8 eV, 284.3 eV, 284.9 eV and 285.8 eV were attributed to C─C, C─N, C─Cl/C─O and C═O, respectively.

XPS results confirmed the successful grafting of PGMA and the introduction of amino group on the surface of the resin.

### 3.2. Effect of pH on adsorption

**Figure 3.**
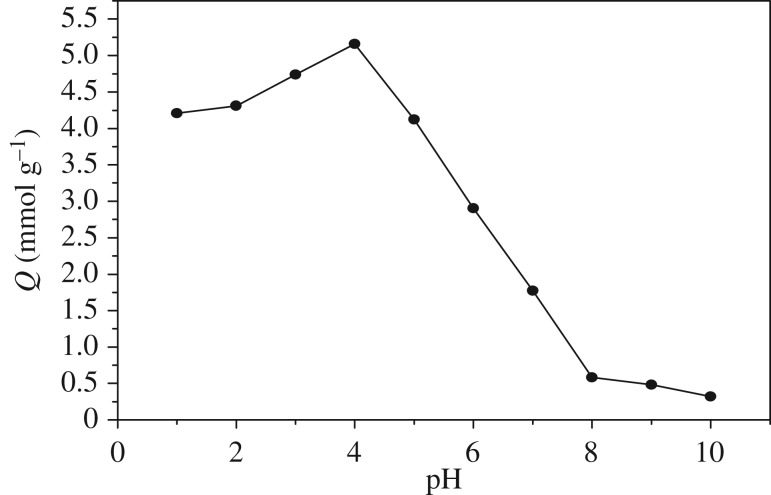
Effect of pH on the adsorption of DETA-PS resin for As(V) (initial concentration: 7 mmol l^−1^; 25°C; adsorbent dose: 0.1 g).

The adsorption of As(V) was examined at different pH, as shown in figure 3. An abrupt increase in the adsorption capacity was observed when pH increased from 1.0 to 4.0, and an abrupt decrease in the adsorption capacity was observed when pH increased from 4.0 to 10.0. When the pH is 10.0, there is no adsorption basically. At pH 2.9, the majority of As(V) exists as H_3_AsO_4_ [14], which cannot be adsorbed by diethylenetriamine resin via electrostatic interaction; so the adsorption capacity is small when the pH value is less than 3.0. From 4.0 to 10.0, the majority of As(V) exists as ${\text{H}}_{2}\text{As}{\text{O}}_{4}^{-}$, $\text{HAs}{\text{O}}_{4}^{2-}$ and $\text{As}{\text{O}}_{4}^{3-}$. As the pH value of the solution increased, the number of protonated amine groups decreased, leading to the decrease of adsorption capacity for As(V).

### 3.3. Adsorption isotherms

**Figure 4.**
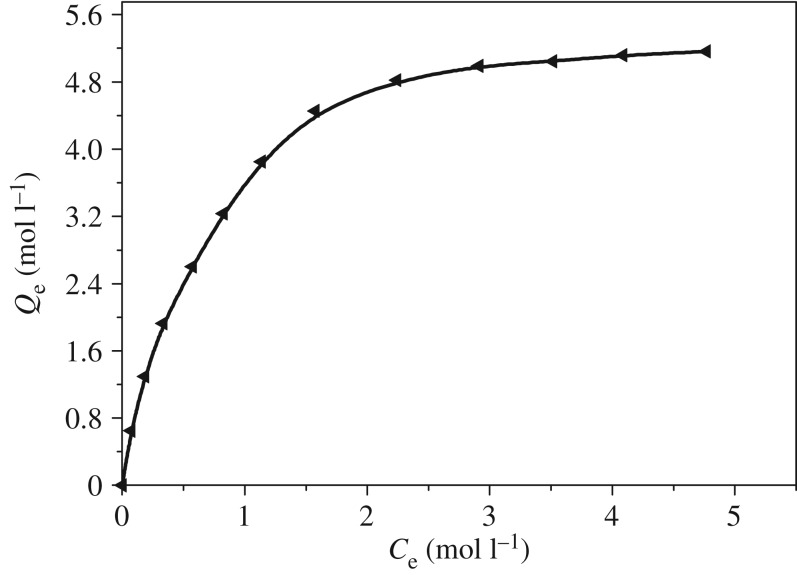
Adsorption isotherm of DETA-PS resin for As(V) (pH: 4.0; 25°C; adsorbent dose: 0.1 g).

Adsorption isotherms are characterized by certain constants, which can explain the surface properties and affinity of the resin, and can also be used to compare the adsorption capacity of metal ions. The adsorption isotherm of diethylenetriamine resin for As(V) at 25°C is presented in figure 4. Two theoretical isotherm models, Langmuir and Freundilich models, are often used to describe and analyse the adsorption isotherm. The Langmuir model is based on the assumption of surface homogeneity such as equally available adsorption sites, monolayer surface coverage and no interaction between adsorbed species, while the Freundlich model assumes that the adsorption occurs on a heterogeneous surface. The adsorption experimental data were analysed by the Langmuir model (3.1) and Freundlich (3.2) model:

3.1 $$\frac{C_{e}}{Q_{e}}=\frac{C_{e}}{Q_{0}}+\frac{1}{Q_{0}b}$$

and

3.2 $$\ln\;Q_{e}=\ln\;K_{F}+\frac{1}{n}\ln\;C_{e},$$

where *Q*_e_ is the adsorption capacity, mmol g^−1^; *C*_e_ is the equilibrium concentration of metal ions, mmol l^−1^; *Q*_0_ is the saturated adsorption capacity, mmol g^−1^; *K*_F_ is an empirical parameter; *n* is the Freundlich constant; *K*_F_ is the binding energy constant reflecting the affinity of the adsorbents to metal ions.

The results are shown in table 1. On comparison of the *R*^2^ values for Freundlich and Langmuir model, we can see that Langmuir equation represented a better fit to the experimental data than Freundlich equation in all cases for the adsorption of As(V). This reflected that the surface of the resin was made up of small homogeneous adsorption patches. According to the Langmuir fitting results, the maximum adsorption capacity (*Q*_m_) was 5.25 mmol g^−1^ for As(V). It was also found that the Langmuir constant (*b*) was 1.30 for As(V).

**Table 1.** Langmuir and Freundlich constants for the adsorption of As(V) on DETA-PS resin at 25°C.

metal ions	Langmuir constants			Freundlich constants		
	*Q*_0_ (mmol g^−1^)	*K*_c_	$R_{L}^{2}$	*K*_F_	1/*n*	$R_{F}^{2}$
As(V)	5.25	73.45	0.99543	3.065	0.6196	0.9566

### 3.4. Adsorption kinetics

**Figure 5.**
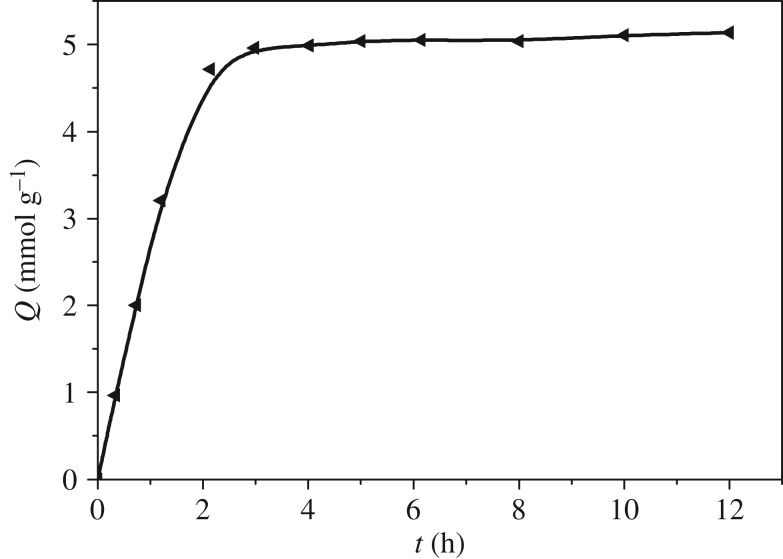
Adsorption kinetics of DETA-PS resin for As(V) (initial concentration: 7 mmol l^−1^; pH: 4.0; 25°C; adsorbent dose: 0.1 g).

The adsorption kinetics reflects the adsorption rate of metal ions, which is an important index to evaluate the adsorption performance of resin. It can be seen from figure 5 that the adsorption amount of As(V) increased sharply with the contact time during the initial 90 min, and reached a maximum at 2 h with the extension of time.

The pseudo-second-order kinetic equation was employed to analyse the adsorption kinetics of three ions. The pseudo-second-order equation is as follows:

3.3 $$\frac{t}{Q_{t}}=\frac{1}{kQ_{e}^{2}}+\frac{1}{Q_{e}}t,$$

where *t* is the adsorption time (min); *k* the adsorption rate constant (min^−1^); and *Q_t_* and *Q*_e_ are the adsorption amount at given time *t* and equilibrium time, respectively.

The data in figure 5 could be converted into plots of *t*/*Q_t_* versus *t*, and good linearity was found with correlation coefficients (*R*^2^) greater than 0.998, indicating that the adsorption kinetics of As(V) on DETA-PS resin followed the pseudo-second-order kinetic model. The adsorption rate constants obtained from the slopes and intercept of the plots were 1.356 g mmol^−1^ h^−1^ for As(V). The kinetic data would be very useful for the fabrication and design of wastewater treatment and noble metal recovery systems.

### 3.5. Comparison with other adsorbents

There are many adsorbents designed for different metal ions reported in the literature, from which representative adsorbents that can adsorb As ions were chosen for comparison with DETA-PS resins. As shown in table 2, DETA-PS resins possessed the advantage mainly in the adsorption capacities. DETA-PS resins had much higher adsorption capacities for As ions than other adsorbents. Higher adsorption capacities can produce higher productivity for water treatment. Therefore, the resin should be of value in water treatment on the basis of sustainability principles.

**Table 2.** Comparison of As(V) adsorption on DETA-PS resins with other adsorbents.

adsorbents	adsorption capacities (mmol g^−1^)	reference
amino-functionalized coffee cellulose adsorbent	0.62	[25]
goethite–P(AAm) composite	0.16	[26]
metal–organic frameworks	1.11	[27]
inorgano–organo–bentonite	0.13	[28]
α-FeOOH@GCA	0.75	[29]
DETA-PS resins	5.25	this work

### 3.6. Adsorption selectivity

The adsorption selectivity is an indispensable factor for evaluating the properties of an adsorbent. In wastewater, the common ions coexisting with As(V) are mainly biologically essential metals, such as Na(I), K(I), Ca(II), Mg(II), Fe(III) and Zn(II). Therefore, the effects of these ions on the adsorption of As(V) on DETA-PS resins were investigated. A total of 100.0 ml of a 7.0 mmol l^−1^ solution of As(V) containing each of the above ions (14.0 mmol l^−1^) was shaken with 0.1 g DETA-PS resins at pH 4.0. As shown in table 3, the influences of Na(I), K(I), Ca(II) and Mg(II) on the adsorption of As(V) can be ignored, but Fe(III) and Zn(II) displayed a minor influence. This phenomenon can be explained based on the hard and soft acids and bases principle. Based on the obtained results, the amino groups had the properties of a soft base. Accordingly, compared to Na(I), K(I), Ca(II) and Mg(II) (hard acids), the metals Fe(III) and Zn(II), being borderline acids, should readily bind with the soft base and can thus interfere with the adsorption of As(V). However, DETA-PS resin prefers adsorbing As(V) rather than Fe(III) and Zn(II). These results illustrated that the resin could selectively adsorb As(V) from wastewater.

**Table 3.** Effect of coexisting metal ions on the adsorption capacity of As(V) (pH 4.0; contact time: 12 h; adsorbent dose: 0.1 g).

coexisting ions	selective coefficients
K^+^	∞
Na^+^	∞
Ca^2+^	∞
Mg^2+^	∞
Fe^3+^	18.2
Zn^2+^	23.7

### 3.7. Adsorption mechanism

**Scheme 2.**
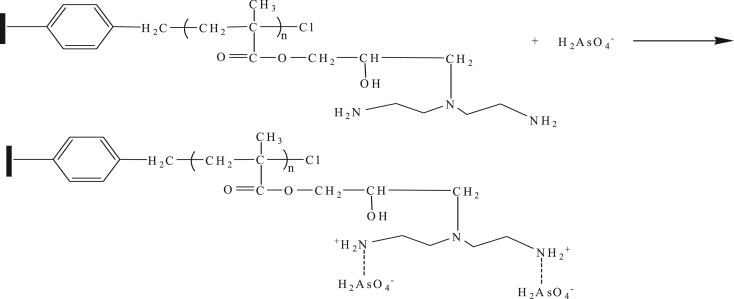
Adsorption mechanism of DETA-PS resin for As(V).

Electrostatic attraction plays an important role in the adsorption process of the protonated amino resin on As(V). Protonated amino groups were responsible for the adsorption of As(V). As shown in scheme 2, As(V) $\left({\text{H}}_{2}\text{As}{\text{O}}_{4}^{-}\right)$ was adsorbed through the electrostatic attraction at pH 4.0.

### 3.8. Reusability of diethylenetriamine-functionalized resin

**Figure 6.**
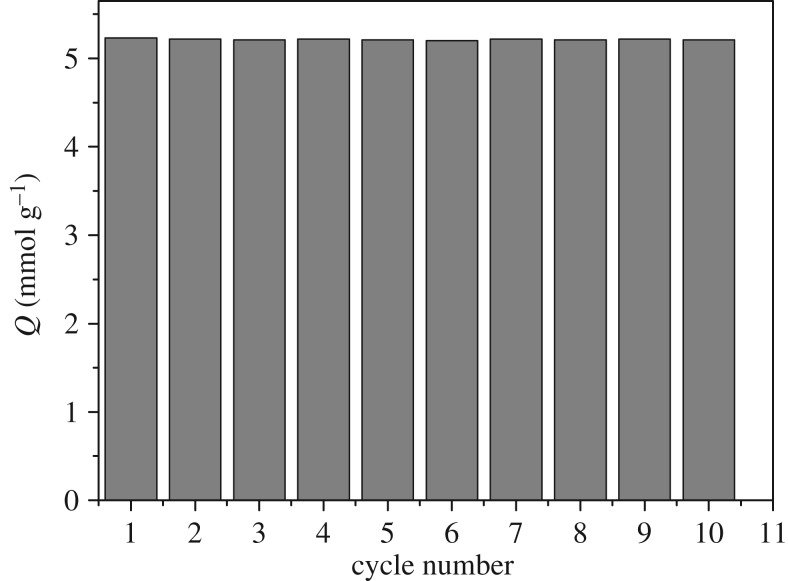
Adsorption and desorption behaviours of As(V) adsorption on DETA-PS resin.

As(V)-loaded resins were placed in 0.1 mol l^−1^NaOH aqueous solution, and the amount of metal ions released in 2 h was determined. Desorption efficiency was found to be generally high (more than 98%). In order to show the reusability of the resin, adsorption–desorption cycles of metal ions were repeated ten times, as shown in figure 6. The adsorption capacity of the resin is reduced by only 2.56% after 10 adsorption–desorption cycles, indicating that adsorption capacity rarely changed during adsorption–desorption operations.

## 4. Conclusion

A new kind of DETA-PS resin was successfully prepared via SI-ATRP and amination reaction. The adsorption capacity of As(V) reached 5.25 mmol g^−1^ at pH 4.0, which is accounted for by the Langmuir adsorption model, and accords with the pseudo-second-order adsorption kinetic equation. Competition from common coexisting ions, such as Na(I), K(I), Ca(II) and Mg(II), was negligible, whereas coexisting Fe(III) and Zn(II) ions displayed only a minor influence, which illustrated the selective adsorption of As(V) from wastewater. No significant change was observed in adsorption capacity after ten adsorption–desorption cycles, indicating that the new resin has a promising application in the treatment of heavy-metal-containing wastewater.

## Data accessibility

This article has no additional data.
